# Interleukins Associated with Breast Cancer

**DOI:** 10.7759/cureus.3549

**Published:** 2018-11-05

**Authors:** Zacharias Fasoulakis, George Kolios, Valentinos Papamanolis, Emmanuel N Kontomanolis

**Affiliations:** 1 Obstetrics and Gynecology, National and Kapodistrian University, Athens, GRC; 2 Pharmacology, Democritus University of Thrace, University Hospital of Alexandroupolis, Alexandroupolis, GRC; 3 Obstetrics and Gynecology, General Hospital of Korinth, Korinth, GRC; 4 Obstetrics and Gynecology, Democritus University of Thrace, University Hospital of Alexandroupolis, Alexandroupolis, GRC

**Keywords:** cytokines, breast cancer, immunotherapy, interleukin

## Abstract

A tumor consists of a group of cells with abnormal growth, capable of acquiring unique characteristics that provide them with the ability to display mercurial migration patterns, adapting to microenvironments and their chemical and physical factors. Interleukins are small proteins secreted mainly by CD3+ and CD4+ T lymphocytes that mediate the "essential for cancer progression" interactions between cells. Interleukins are implicated in both the development and differentiation of different cells (NK, B, and T leukocytes) and, in general, play a major role in many diseases, including breast cancer, due to their unique participation in systemic inflammation and immune system modulation. During the past decade, interleukins proved to be decisive for future immunotherapy, predisposing a more reliable treatment with fewer side effects on normal proliferating cells. The aim of this review is to provide an overview of the role of interleukins implicated in breast cancer progression.

## Introduction and background

Breast cancer represents the second main cause of death caused by cancer in America while it is the leading diagnosed cancer in the United States and accounts for approximately 30% of all new cases of women diagnosed with cancer. Even though breast tumor development still remains partly clarified, several theories have emerged over the past years considering the initiating point. Virchow R noted, in 1863, the presence of leukocytes in neoplastic tissues and was the first to describe a potential correlation between cancer development and inflammation. Over the past decades, inflammation has proved to play a critical role in tumor development and progression while many of the major molecular mechanisms are now revealed, highlighting the key role of cytokines, and especially interleukins (ILs), in the breast cancer initiation, migration, and progression pathway [1]. The aim of this study is to review the role of ILs in breast cancer.

## Review

Interleukins correlated with breast cancer

**Figure 1:**
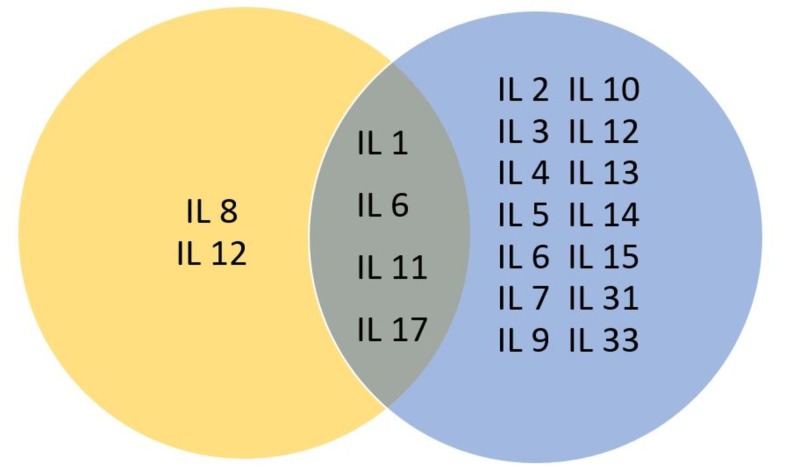
Interleukins involved in acute and chronic inflammatory responses

Cytokines are biomolecules whose biological properties suggest a key role in infections, hematopoiesis, and homeostasis, revealing their multifunctional role that controls response against infectious diseases and even tumorigenesis by controlling tissue renewal, cellular sprouting, and growth. ILs are secretory immunomodulatory proteins that belong to the superfamily of cytokines and, as cytokines, present complex immunological functions. The main objective of ILs is to mediate intercellular communication in the immune system, including cell migration, proliferation, maturation, and adhesion, which, as mentioned, plays a vital role in the inflammatory response [2]. Interleukins are involved in both acute and chronic inflammatory responses (Figure 1). They act as a response to the stimulation of specific receptors expressed on the cell surface, activating a particular signaling pathway each time. To date, about 38 different interleukins have been identified, each binding to a unique type of receptor, having a specific origin, structure, and properties while many of them are being reported to be present and participating in the induction and propagation of breast cancer (Table 1).

**Table 1:** Source cells and the main function of interleukins implicated in breast cancer LAK: lymphokine-activated killer cell; TNF: tumor necrosis factor; Th cell: T helper cell; NK cell: natural killer cell

Interleukin	Source cell	Main function and act in breast cancer
IL-1	Epithelial cells; Endothelial cells; Neutrophils; Mononuclear phagocytes	Proinflammatory cytokine; Induction of Th17 cells
IL-2	Th1-cells	Immunoregulatory cytokine; Activates NK cells and monocytes; Main growth factor for B and T lymphocytes
IL-4	Th2-cell; Mast cells; Basophils	Control of parasitic infections; Antiinflammatory effect by inhibition of interleukin-1; TNF and IL-6 production by monocytes; Inhibition of the Th17 pathway; Antibody-mediated immunity
IL-6	Macrophages; T cells; Fibroblasts	Promotes tissue invasion / epithelial to mesenchymal transition; Induction of acute-phase proteins; Effects on B cells; Induction of Th17 cells
IL-7	Stromal Cells; Thymus	Induces Type 1 immune response; Increases CD8+ specific cytotoxicity; Induces NK and LAK cell
IL-8	Macrophages; Neutrophils; Endothelial cells; Fibroblasts	Neutrophil chemoattractant and activator
IL-10	B cells; T cells; Monocytes	Promotes angiogenesis and tissue invasion; Inhibits mechanisms of growth and metastasis suspension.
IL-11	Fibroblasts; Stromal cells of bone marrow	Hematopoietic growth factor; Stimulates thrombopoiesis and growth and differentiation of bone marrow cells (that differentiate into macrophages)
IL-13	Th2 cells; CD8+ T cells; Mast cells; Eosinophils; Basophils	Inhibits proinflammatory cytokines; Modulates macrophage function; Attenuates interaction with activated endothelial surfaces
IL-17	Th17 cells	Control of extracellular pathogens; Synergy with TNF and interleukin-1; Proinflammatory cytokine; Induction of matrix destruction
IL-19	B cells; Monocytes	Increases growth, proliferation, and cancer progression
IL-21	Th17 cells	Amplification of the Th17 pathway in autocrine fashion
IL-23	M1 dendritic cells; T helper 1 cells; Monocytes	Promotes inflammation; Th17 expansion and stabilization
IL-32	Keratinocytes	Proinflammatory cytokine
IL-33	T helper 2 cells; Mast cells; Innate helper 2 cells	Decreases apoptosis of myeloid-derived suppressor cells

Interleukin 1

Interleukin-1 (IL-1) represents a group of 17-20 kilodalton (kDa) cytokines that are characterized by a variety of biological functions. The main representatives are the proinflammatory cytokines IL-1a and IL-1b, interleukin-1 receptor antagonist (IL-1ra), all implicated with the initiation and progress of inflammatory processes. Their activity is determined by two neighboring genes identified on chromosome 2: IL1A and IL1B [3]. Many cancer types reveal a high expression of IL-1 while a virulent tumor phenotype is associated with many cancer types with high IL-1 expression and poor prognosis [4-6]. A possible autocrine pathway is suspected to exist involving IL-1β and the activation of the NF-kB pathway. Metastatic breast cancer lines secrete IL-1β with the possible existence of other factors, which act on mesenchymal stem cells (MSCs). The MSCs, with the assistance of chemokines, will strongly influence the metastatic-invasive potential of the breast neoplastic cells. MSCs exhibit pro- or anti-tumoral activities within the tumor microenvironment; they produce chemokines that affect chemotaxis and other parameters of the cells’ behavior. Chemokines behave as chemo-attractants and drive cells towards areas of higher concentration of the factor [7].

IL-1 induces the expression of metastatic genes such as matrix metalloproteinases (MMP) by generating growth factors and angiogenic proteins produced by nearby cells, promoting cancer development via neovascularization and metastasis. Perrier et al. presented in 2009 a model that reveals the possible regulation mechanism of IL-1 to breast cancer. According to their results, proliferation, migration, and invasion in breast cancer are all affected by both agonists (IL-1, leptin) and antagonists (IL-1ra, adiponectin) expressed in mammary adipose cells acting through specific receptors in both an autocrine and a paracrine way on mammary tumour cells, regulating the production of growth factors and epithelial-derived and angiogenic proteins, stimulating the invasion and proliferation of other cancer cells [8]. Filippi et al. also supported that IL-1 contributes to breast cancer metastases. They reported that hypoxia promoted the triple negative breast cancer cell line MDA-MB-231 cell migration along with hypoxia-inducible factor 1-alpha (HIF-1α) accumulation and upregulation of chemokine (C-X-C motif) receptor 1 (CXCR1) [9]. Holler et al. investigated the expression of IL-1 in breast cancer by blocking IL-1R signaling. The authors reported that IL1 has a functional role in breast cancer growth and bone metastasis [10].

Interleukin 2

The lymphocytotrophic 15.5 kDa glycoprotein interleukin-2 (IL-2) is located in the plasma membrane and expressed in normal tissues, the endothelial cells, and the intestinal epithelium; this unique interleukin demonstrates an active role in the growth sprouting and differentiation of the T and B cellular groups and non-lymphoid cells and promotes the cytolytic properties of the natural cells (NK cells). Biochemically, the IL-2 receptor complex (IL-2R) encompasses three subunits (α, β, γ) deciphered by different and unrelated genes. It is synthesized by CD4+ T helper (Th) lymphocytes, previously known as the T cell growth factor. The production of IL-2 was first connected to breast cancer progression in patients treated for breast cancer who had a relapse percentage of 4.7% if the IL-2 plasmatic level was normal while the relapse percentage increased to 33.3% if IL-2 was low after a 10-12 month follow-up [11]. Garcia-Tuñón et al. showed in clinical studies that the IL-2 expression, including its three receptors chains, was more prominent in breast-infiltrating tumors compared to the in situ surgical samples. This in accordance with the knowledge that the infiltrating neoplasms are characterized by higher aggression [12-13]. Xiao-Bo et al. reported that polymorphism in the IL-2 gene was connected to increased rates of breast cancer and could be used as a marker for the prognosis of the disease while Muraro et al. studied patients with human epidermal growth factor receptor 2 (HER-2) - overexpressing and negative - in locally advanced breast cancer and reported that IL-2 can influence the initiation and development of cancer in HER-2 patients since they proved to carry considerably lower amounts of IL-2 [14-15].

Interleukin 4

Interleukin 4 (IL-4) acts on B lymphocytes, monocytes, dendritic cells, and fibroblasts, determining allergic responses, and has proven to have both antitumor and anti-inflammatory effects. Janus kinase/signal transducers and activators of transcription (JAKs and STAT) pathways mediate IL-4 transducing signals while its expression and production are restricted to activated T lymphocytes, mast cells, and basophils [16]. The implication of IL-4 in the pathogenesis of breast cancer development and resistance to apoptosis and local metastasis is reported in several studies [17]. The IL-4 receptor is significantly expressed in breast cancer while IL4R is compulsory for the actions of IL-4 on cancer cells [18]. Nagai and Toi reported that IL-4 is responsible for the regulation of the enzymes involved in the synthesis of estrogen-promoting apoptosis in cultured breast cancer cells. Gaggianesi et al. studied the impact of blocking IL-4 with the IL4Rα antagonist IL4DM in mammary gland tumors, reporting the inhibition of cancer cell proliferation, invasion, and growth that was achieved by downregulating mitogen-activated protein kinase (MAPK) pathway activity [19]. IL-4 and the IL-4/IL-4R signaling axis are also one of the main subjects studied, considering the therapeutic inhibition of interleukins, since IL-4 protects tumor cells from CD95- and chemotherapy-induced apoptosis through the upregulation of antiapoptotic proteins, IL-4R has proven to directly promote mammary tumor metastasis and that blocking IL-4 protects against the macrophage-mediated radioresistance of inflammatory breast cancer [20-21].

Interleukin 6

Interleukin-6 (IL-6) is a 26 K.D cytokine synthesized by vascular endothelial cells and mononuclear phagocytes fibroblasts encountered in bladder and cervical cancer. It is secreted by actions induced by IL-1 and TNF and acts primarily on hepatocytes and B cells. Interleukin-6, as the major inducer of the inflammatory response, carries the principal role in the pathology and angiogenesis of cancer. Neoplastic cells, when triggered by the IL-6 presence, show the ability to intrude the extracellular matrix (ECM) and significant drug resistance. Carcinoma cells selected from renal, bladder, cervical and breast cell lines proved to potentially express IL-6. The IL-6 receptor is subsequently expressed on prostate, ovarian, renal, and breast cellular membranes. IL-6 shows its multifunctional role in many ways. It is secreted by different tumors and its proliferation is interrelated with an auto-paracrine mode of neoplasm stimulation. IL-6 has the ability to upregulate any anti-apoptotic proteins or induce the conglomeration of pro-angiogenic cytokines. Furthermore, IL-6 triggers the production of the vascular endothelial growth factor (VEGF) in cell lines. Oncology patients quite often suffer from chemotherapy-related thrombocytopenia. IL-6 serves in these reported cases as a potent thrombopoietic cytokine. VEGF detection in the human megakaryoblastic leukemia cell line, MEG-01, is regulated by IL-6 (unpolished data). It is highly probable for IL-6 to act as an angiogenic agent; it regulates the concentration of the VEGF molecule in the platelets plus the promoted endothelial cell multiplication. The ongoing neoplastic growth process is suggested from the IL-6 serum levels. It is very likely for the bad survival rate to be connected with serum IL-6. Xian-Peng reported that IL-6 was responsible for maintaining the growth of human breast adenocarcinoma cell line MCF-7 when cultured with human IL-6 and IL-6 soluble receptor [22]. Moreover, aiming breast cancer cells with IL-6 inhibits proliferation in estrogen receptor (ER) positive cells, however, the main role of IL-6 in breast cancer considers the prognostic use with elevated circulating IL-6 levels being correlated with a poor prognosis in many studies [23].

Interleukin 7

Interleukin-7 is a ~25kDa glycoprotein regulated by a gene located on chromosome 8q12-13. IL-7 is produced by stromal cells in the bone marrow and the thymus-stimulating proliferation of both pre-pro B cells -together with hepatocyte growth factor (HGF) in bone marrow and the precursors of T cells (in the thymus). IL-7 can bind to a cell surface receptor (IL-7R) composed of two chains, the γc chain and the IL-7Rα chain, which maps to chromosome 5p13. IL-7 can bind to a cell surface receptor (IL-7R) composed of two chains, the γc chain and the IL-7Rα chain, which maps to chromosome 5p13. Expressed on endothelial cells, IL-7 affects the formation of new lymphatic vessels. Reports of the latest decade indicated that the aberrant expression of IL-7 and IL-7Ra polymorphism play distinct roles in breast carcinogenesis according to the molecular subtype [24]. Recently, IL-7 was proposed to play a role in the pathogenesis of mammary gland carcinogenesis, promoting the survival and development of cancer cells in culture, with its expression being connected to poor prognosis in human samples. Al-Rawi et al. reported that IL-7 induced the growth of breast cancer cells in vitro through a wortmannin-sensitive pathway [25]. Boesch et al. published a study in 2017 where IL-7-expressing cancer-associated fibroblasts promoted breast tumor growth and provided critical niches for the maintenance of breast cancer stemness while CXCL12 was identified as an important niche factor in Il7-expressing cancer-associated fibroblasts (CAFs), indicating that the stromal cell-derived factor 1/cluster of differentiation 184 CXCL12/CXCR4 pathway may serve as a therapeutic anti-cancer stem cell (anti-CSC) target [26].

Interleukin 8

Interleukin 8 (IL-8, neutrophil-activating protein-1) is an 8 KD protein of 72 residues produced by macrophages and endothelial cells. It demonstrates a powerful chemotactic effect on T-lymphocytes and neutrophils and upregulates the binding properties of leucocyte adhesion receptor CD11b/CD18. IL-8 demonstrates antiviral immunomodulatory (they affect an immune response either positively or negatively) and antiproliferative properties. It participates in the inflammation and migration of cells. Being a member of the CXC chemokine group, IL-8 is potentially produced and expressed in normal and neoplastic human cell lines, i.e., breast cancer, ovarian cancer, prostate cancer, thyroid cancer, and many others. Nitric oxide (NO) is proved to be a principal, intracellular second messenger to regulate IL-8 gene expression in concert with hypoxia and anoxia; NO is produced from L-arginine by the enzyme NO synthase in the presence of O2 and co-factors [23].

IL-8 presents both autocrine and paracrine identity showing a tumor-inducing face and a significant potential in the role of a predictable and prognostic neoplastic index. It is expressed in ER- breast cancers, increasing invasiveness and metastatic potential in both ER- and ER+ breast cancer subjects. IL-8 participates in inflammatory-related clinical conditions; its source of secretion are the fibroblasts, endothelial cells, and tumor cells. IL-8 possesses two receptors, the IL-8RA and the IL-8RB. The noteworthy expression of IL-8 represents the invasive dynamic of breast cancer lines; even higher serum levels of IL-8 indicate seeding of the breast neoplastic cells [27].

Breast tissue of normal and neoplastic origin is occupied with a variety of cytokines. Green et al. investigated a group of messenger RNA (mRNA) transcripts for cytokines with the method of reverse transcriptase-linked polymerase chain reaction (RT-PCR). The following cytokines were screened: interleukin (IL)-a, IL-1β, IL-2, IL-3, IL-4, IL-5, IL-6, IL-7, IL-8, tumor necrosis factor (TNF)-β, TNF-β, and interferon (IFN)-γ. The detailed analysis revealed increased titers of IL-8 only. What is significant in this laboratory analysis is the complete absence of the above-mentioned cytokines; these cytokines are parts of cells of the immune system [28]. In another study, 20 normal and 73 neoplastic breast tissues were examined considering the mRNA profile of 13 cytokines. The MCF-7 and MDA-435 cellular lines having tumorigenic and metastatic properties were tested. The tumor masses occupied with the MCF-7 cell type did not give any metastasis, whilst MDA-435 metastasized to a great extent in about 50% of the cases. This is in accordance with the initial assumption that the MDA-435 cellular line, being the highest secreting IL-8 line, invaded the distant anatomical sites more aggressively compared to the MCF-7 neoplasms. Among the cytokines studied, IL-8 was detected to have the highest concentrations [29].

Interleukin 10

Interleukin 10 IL-10 is a pleiotropic cytokine with a molecular weight of approximately 18 kDa (monomer). IL-10 is produced by TH0, TH1, TH2, Treg, cytotoxic T cells, mast cells, and activated monocytes and was originally called the cytokine synthesis inhibitory factor due to its ability to inhibit the production of certain cytokines. IL-10 represents the best-studied and most well-known anti-inflammatory cytokine in general [30]. Many studies have reported the implication of IL-10 in mammary carcinogenesis by revealing the high IL-10 mRNA expression of breast tumor cells. Moreover, its multifunctional role, including both immunosuppressive and antiangiogenic functions, is proven to play varied roles in the pathogenesis, progression, metastasis, and development of breast cancer [31-34]. IL-10 is also a good prognosticator of disease-free survival in non-basal, non-triple-negative, ER-positive, and progesterone (PR)-positive breast cancer subtypes [35].

Interleukin 11

Interleukin-11 (IL-11) is a 65-85 kDa protein produced by fibroblast and stromal cells in the bone marrow, which belongs to a large group of cytokine ligands encompassing IL-6, IL-11, leukemia inhibitory factor (LIF), IL-31, IL-27, and cardiotrophin-like cytokine (CLC). Interleukin-11 is accompanied by its transmembrane receptor IL-11 receptor-alpha (IL-11Rα) and stimulates the proliferation of the breast neoplastic cells with the simultaneous growth of the primary cancer cells and the migration of the cancer cells into distant organs. In addition, IL-11 expression in breast cancer tissue correlates with a high histological grade along with poor patient survival [36]. Mengyao et al. reported the correlation of IL-11 expression with Her-2 and Ki-67 expression in breast cancer, the correlation of IL-11Ra expression levels with ER, PR, and Her-2 expression while the co-expression of IL-11 and IL-11Ra was correlated with microvessel density and angiogenesis in breast cancer patients [37].

Interleukin 13

Interleukin-13 (IL-13) is a 12-kDa cytokine, primarily produced by TH2 cells but also secreted by other T helper cell subsets: CD8+ T cells, mast cells, eosinophils, and basophils. IL-13 is, in general, homologous to IL-4, sharing many of its biologic activities on mononuclear phagocytic cells, endothelial cells, epithelial cells, and B cells, even though the responses of cells to IL-13 are smaller in magnitude. IL-13 is proven to be overexpressed in breast cancer [38]. The involvement of IL-13 in breast carcinogenesis has also been proved in many recent studies. Breast cancer tumors are infiltrated with CD4+ T cells secreting IFN-γ and IL-13 while breast cancer instructs dendritic cells to prime IL-13-secreting CD4+ T cells that facilitate tumor development. Moreover, the IL-13 expression level is inversely correlated to ER and PR, revealing a possible involvement in the aggressiveness of ER- breast tumors, and, thus, it is being considered a potential prognostic factor for breast malignancy, with studies testing the results derived by its’ inhibition [33].

Interleukin 17

Interleukin 17 IL-17 is a pro-inflammatory produced by CD4 Th17 and CD8 Tc17 cells, presenting the most complex synthesis among all the cytokines. IL-17 is mainly produced by Th17 cells. The IL-17 family contains six members designated as IL-17A through IL-17F. IL-17 reveals a proinflammatory action on a variety of cell types, inducing prostaglandins, nitric oxide, cytokines, and chemokines. IL-17 cytokines have a protumor effect either specifically on tumor cells or in a collateral way by affecting the patient's antitumor reaction and by causing microenvironmental changes that aggravate the invasive and metastatic profile of the disease [39]. Chen et al. reported in 2013 the correlation between elevated rates of IL-17-producing cells with a high histological grade, negative ER/PR status, and triple-negative molecular subtypes segregated by immunoprofiles. Elevated expression levels of IL-17-producing cells in the mammary gland tumor microenvironment is correlated to poor prognostic factor for staging, overall, and disease-free survival [40].

Interleukin 19

Interleukin 19 (IL-19) is a member of the IL-10 family, secreted by monocytes. The IL-19 protein induces the assembly and expression of fibronectin (FN), cell multiplication, and metastasis in breast cancer cells. Fibronectin is the binding protein in the stroma that promotes neoplastic cells in lung metastasis in breast cancer. There is a combination of interactions among cells and the matrix, a secretion of cytokines that propagates distant invasion. Peptide and antibody inhibitors of fibronectin are proving to be effective inhibitors of metastasis and are potentially important reagents for the study and control of cancer. The expression of IL-19 is upregulated by the presence of granulocyte, macrophage colony-stimulating factor (GM-CSF), lipopolysaccharide (LPS), interleukin 6 (IL-6), and TNF-a. The genetic profile of IL-1β, IL-6, transforming growth factor (TGF)-β, MMP2, MMP9, and CXCR4 is modulated by IL-19. The structure at the cellular level is determined by cell-cell and cell-matrix interactions; each of these interactions is a requirement for metastasis [41-42]. Being implicated in breast pathogenesis, IL-19 has an autocrine effect in mammary gland cells and represents a major prognostic factor for different types of tumors, including breast cancer. IL-19 promotes proliferation and migration directly while secondarily, it provides a microenvironment for tumor development, with high IL-19 expression levels being associated with a poor clinical outcome for breast cancer patients [41-42]. IL-19 is reported to directly affect breast cancer cell proliferation, migration, and tumor progression by inducing the expression of cytokines and chemokines while the expression levels are connected to advanced stages, higher mitotic rates, and metastasis. IL-19 expression levels have proven to predict worse disease-specific survival and metastasis-free survival, and it is believed that IL-19 acts primarily as a local mediator in the microenvironment that affects breast cancer cells and that acts in an autocrine manner in breast cancer [41].

Interleukin 21

Interleukin 21 (IL-21) is encoded by gene IL21 and detected in chromosome locus 4q26-q27, with a domain of 131-amino-acid four-helix-bundle cytokine sequence, structurally related to IL-15 and IL-2. The cluster of differentiation 4 (CD4+) and the natural killer T cells are the main sources of secretion. IL-21 is involved in a diversity of biological and immunological mechanisms through its cognate receptor IL21R. Li-Wang et al. demonstrated, with the use of a reverse-transcriptase-polymerase chain reaction (RT-PCR), western blotting, and sequence analysis of triple-negative breast cancer cellular lines (MDA-231), the differential expressions of IL-21R in breast cancer tissues. IL-21 proved to promote proliferation, migration, and invasion of MDA-231 cells, which can even enhance the invasive properties of tumor cells [43].

Interleukin 23

Interleukin 23 (IL-23) is a member of the IL-6 family of heterodimeric cytokines composed of two disulfide-linked polypeptide chains, p19 and IL-12 p40. IL-23 is closely related in structure to IL-12. IL-23 induces the differentiation of naive CD4+ T cells into Th17 cells and represents an important therapeutic target for the treatment of many chronic immune-inflammatory disorders. In 2012, Gangemi et al. presented a study where the authors reported a negative prognostic correlation of increased IL-23 levels in the overall survival of patients with breast cancer [44]. Sheng et al. unveiled, even more, the connection of IL-23 to breast cancer by studying the expression levels of interleukin (IL)‑23/IL‑23 receptor (R) gene reporting increased results in breast cancer tissues, correlating IL‑23 and IL‑23R expression levels with the patients' tumor size, stage, and metastasis [45].

Interleukin 32

Interleukin (IL-32, NK4) is a novel cytokine originally isolated from activated T cells. IL-32 behaves as a multifaceted cytokine involving autoimmune diseases, infections, and neoplasia, inducing inflammation in the tumor environment and quite often blocking viral multiplication while it has a vital role in endothelial functions and angiogenesis. IL-32 expression is associated with increased invasion and migration in breast tumors and exerts modulatory effects on the growth and survival of breast cancer cells. Wang et al. performed laboratory studies and underscored the proliferative potential of IL-32 on neoplastic breast cells reporting that IL-32 has an effect on cancer cells blocking their apoptotic characteristic [46-47].

Interleukin 33

Interleukin-33 (IL-33) is a member of the IL-1 superfamily of cytokines that induces helper T cells, mast cells, eosinophils and basophils to produce type 2 cytokines [48]. IL-33 and its receptor ST-2, mediates its pro-inflammatory-protective identity, through the IL-33/ST-2 axis. The ST-2 receptor presents the full-length membrane type (ST-2L) and the soluble subtype (sST2). Liu et al. confirmed the expression of IL-33 in breast cancer samples in comparison to normal tissues, the overexpression of HER-2, significant lymph node detection including a positive medical record for mammary gland malignancy. Jafarzadeh et al. evaluated the serum levels of IL-32 and reported that the IL-33 levels and IL-33/IL-12 ratio in patients with stage IV breast cancer were significantly higher compared to other stages and controls (P<0.0001 and P<0.001, respectively), revealing the contribution to breast cancer progression by the imbalance occurred in helper T (Th) cell responses Th1/Th2 [49-50].

## Conclusions

Interleukins have been assigned a vital role in the homeostasis of normal tissues and cells, as well as in the induction and propagation of pathologic mechanisms and especially in breast cancer. In order to investigate the role of interleukins in breast pathology, different methods have been tested and many studies have been performed during the past decades. Interleukins are now proven to determine the establishment, proliferation, and migration of breast cancer, affecting the invasive and metastatic profile of the disease, inducing angiogenesis and tumor growth, acting as prognostic factors and even determining the survival rates of breast cancer. Even though recent data provide valuable information on the pathogenic role of interleukins proven to participate in breast cancer progression and development, many interleukins implicated in breast cancer still require additional research in order to further exploit their role and overall implication and to test their potential therapeutic use in breast cancer.
